# Association between home and community-based services utilization and self-rated health among Chinese older adults with chronic diseases: evidence from the 2018 China Health and Retirement Longitudinal Study

**DOI:** 10.1186/s12889-023-17535-1

**Published:** 2024-01-08

**Authors:** Tingke Xu, Zishuo Huang, Bingzhen Li, Haojie Jin, Jiayun Zhang, Huiting Yang, Yucheng Huang, Xiangyang Zhang, Chun Chen

**Affiliations:** 1 School of Public Health and Management, Wenzhou Medical University, Wenzhou, Zhejiang, 325035 China; 2 School of Innovation and Entrepreneurship, Wenzhou Medical University, Wenzhou, Zhejiang 325035 China; 3 The 2nd School of Medicine, Wenzhou Medical University, Wenzhou, Zhejiang, 325035 China; 4 First Affiliated Hospital of Wenzhou Medical University, Wenzhou, Zhejiang 325000 China; 5 Institute for County Chronic Disease Health Management Research, Wenzhou Medical University, Wenzhou, Zhejiang 325000 China

**Keywords:** Home and community-based services, Self-rated health, Older adults with chronic diseases, Multilevel analysis

## Abstract

**Background:**

As global aging intensifies, older adults with chronic diseases are of increasing concern. Home and community-based services (HCBSs) have been proven to promote self-rated health (SRH) in older adults, but no research explored the associations between the use of overall HCBSs, three different types of HCBSs (health care, daily care, and social support services) and SRH among older adults with chronic diseases. Consequently, this study applies a national publicly available database to examine these associations among older adults with chronic diseases.

**Methods:**

8,623 older adults with chronic diseases (≥ 60 years old) were included in this study. SRH was evaluated applying a concise question with a 1 − 5 scale. HCBSs utilization was assessed through the question, “What kind of HCBSs were used in the community?”. Univariate general linear regression models aimed to compare the mean values of SRH in terms of HCBSs utilization in each group. This study is a cross-sectional study design and the relationship between HCBSs utilization and SRH was assessed by multilevel linear regression.

**Results:**

The mean score for SRH among the respondents was 3.19, of whom 20.55% used one or more HCBSs, 19.47% utilized health care services, 2.44% utilized social support services, and only 0.55% utilized daily care services. The use of HCBSs was found to be linked to SRH among older adults with chronic diseases (β = 0.085, SE = 0.025, *p* < 0.001). SRH among older adults with chronic diseases was strongly linked to the use of health care and social support services (β = 0.068, SE = 0.025, *p* < 0.001; β = 0.239, SE = 0.063, *p* < 0.001, respectively). However, there was no significant association between the use of daily care services and SRH among older adults with chronic diseases.

**Conclusion:**

This study revealed that HCBSs utilization was positively and significantly linked to SRH in Chinese older adults with chronic diseases. Furthermore, this study supposes the low utilization of social support and daily care services may be due to a mismatch between supply and demand. The government should offer the targeted HCBSs for older adults with chronic diseases according to their unique features to enhance their health status.

**Supplementary Information:**

The online version contains supplementary material available at 10.1186/s12889-023-17535-1.

## Introduction

As the global demographic has transitioned, aging phenomenon has become a prominent social problem, especially in China [1]. In terms of the seventh national census, 18.7% of China’s population is over 60 years of age, at more than 260 million people, and older adults will become the fastest-growing group in the next 30 years [2]. With this aging trend, World Health Organization (WHO) has noted the burdens of disease have shifted from maternal, child and communicable diseases to noncommunicable diseases [3].

Noncommunicable diseases, which are also regarded as chronic diseases, tend to be related to long duration. They kill 41 million people per year globally, accounting for 74% of all deaths, with older adults featuring prominently [4]. Chronic diseases in older adults may also result in higher rates of disability compared to the general population [5–7]. The latest data furnished by the National Health and Wellness Commission in 2019 showed over 180 million older adults with chronic diseases in China [8]. Moreover, the cost of chronic diseases accounted for over 80% of all disability-adjusted life years in China and formed the largest disease burden in the country [9]. Hence, the care issues of chronically ill older adults in China require more attention.

In parallel with the demographic and health transition, China is also going through a huge social change [10]. Shifts in family structures, urbanization and an expanding population of ‘empty nesters’ older adults are threatening the conventional family-based model of care for chronically ill older adults [11]. Furthermore, chronically ill older adults might be unwilling to be moved into institution because the traditional institutional care model has a limited number of older adults to accommodate and a relatively high cost of care [12]. In order to tackle these issues and allow older adults with chronic diseases to remain where they are, since 2008, the Chinese government has encouraged home and community-based services (HCBSs) nationwide, mainly including health care, daily care, and social support services [13]. HCBSs play a vital role in promoting older adults’ life satisfaction, preventing them from being placed to nursing home, and reducing the burden of diseases [14]. Older adults with chronic diseases are the target vulnerable population of HCBSs policy, being regarded as one of the priority clients of HCBSs [15]. HCBSs specialized services to provide socialized care to assist chronically ill older adults to manage chronic conditions independently at home and in the community [15–18].

Self-rated health (SRH) has been widely recognized as one of the most commonly applied variables in geriatric and health research [19]. Containing physical, mental, and social factors, SRH is a comprehensive measurable index reflecting older adults’ subjective and objective health status [20]. It has been proven that SRH predicts individuals’ quality of life [21], ability to perform activities of daily living [22], and mortality [20], being considered as one of the most significant health indicators by WHO [23]. In recent years, existing studies have explored the role of HCBSs in older adults’ SRH [24]. For instance, Tetyana et al. examined the ethnic disparities in the relationship between the use of HCBSs and SRH among American older adults [25]. Jessica et al. demonstrated a correlation between perceived access to HCBSs and SRH in older adults living in the age-friendly community [26]. Yang et al. have demonstrated the role of different types of HCBSs in SRH among general older adults using data from Shaanxi Province, China [27]. However, no study explored the association between overall HCBSs and different types of HCBSs (health care, daily care, and social support services) utilization and SRH among chronically ill older adults in China. Older adults with chronic diseases are more likely than the general elderly population to have adverse effects that trigger poor SRH due to the loss of work capacity and mobility, increased possibility of depression, and poor health-rated quality of life [28, 29]. Consequently, focusing research on chronically ill older adults is of considerably significance.

It is conceivable that older adults’ SRH has a hierarchical structure, as they are spread across provinces—older adults’ SRH would be affected by individual [30] and by provincial factors, including gross domestic product (GDP) and the number of beds in medical institutions per 10,000 persons in each province [31]. All previous studies have employed regression analysis to test the role of HCBSs in older adults’ SRH while only adjusting individual-level covariates, when the provincial variables could bias these estimations. Therefore, to fill these research gaps, this study uses the China Health and Retirement Longitudinal Study (CHARLS) to examine the relationship between overall HCBSs and different types of HCBSs (health care, daily care, and social support services) utilization and SRH among older adults with chronic diseases adjusting individual and provincial covariates. This study is of utmost significance as it could contribute to policy development on chronic diseases management of older adults in the community.

## Methods

### Study design

This study is a cross-sectional study conducted in the community. Multilevel analysis was applied to explore the relationships between overall HCBSs and different types of HCBSs (health care, daily care, social support services) utilization and SRH among chronically ill older adults after controlling individual and provincial factors, using 2018 CHARLS database.

### Participants

In this study, respondents were excluded if they (1) were aged below 60 years (**8762**), (2) lacked data for HCBSs utilization (**35**), (3) lacked SRH data (**955**), (4) lacked values for the main variables (**33**), or older adults without chronic diseases (**1408**). A total of 19,816 respondents completed the CHARLS in 2018, of whom 8,623 were included in the study.

### Variables

SRH, the dependent variable, was evaluated applying the question, “How would you rate your overall health during the past year?” In terms of the recommendations of WHO [32], SRH scores are divided into the following five options: 1 = very good, 2 = good, 3 = fair, 4 = bad, and 5 = very bad. SRH was listed as a continuum variable, ranging from 1 to 5, with a higher score reflecting worse SRH.

The use of HCBSs was assessed by asking respondents, “Have you ever received the following home and community care services?” The possible responses were as follows: (a) day care centers, nursing homes, senior dining tables, etc.; (b) regular physical examinations; (c) onsite visits; (d) family beds; (e) community nursing; (f) health management; (g) entertainment. Of these, (a) was classified as a daily care service, (b), (c), (d), (e), and (f) were classified as health care services, and (g) was classified as a social support service. We separated older adults with chronic diseases into two groups given whether they used any HCBSs. Those who used one or more HCBSs were placed in the HCBSs use group, and those who did not were allocated to the non-HCBSs use group. The division of the three service categories (daily care, healthcare, and social support services) into variables was consistent across the HCBSs’.

Covariates are comprised of individual and provincial variables [33–36]. The individual variables contained age (60-, 70-, and 80-), gender (male and female), marital status (partnered and single), education level (illiterate, elementary school, middle school, and high school or higher), residence (urban, urban-rural continuum, and rural), income (yes or no), health insurance (yes or no), smoking (yes or no), drinking(yes or no), exercising(yes or no). The provinces included in the study were ranked from the lowest to the highest in terms of GDP per capita and the number of beds in medical institutions per 10,000 persons in each province which placed in the first (Q1), second (Q2), third (Q3), or fourth (Q4) quartile.

### Data sources

This study applied the data from the 2018 CHARLS, a survey that covers numerous topics in relation to the socioeconomic status and health conditions of Chinese middle-aged and older adults [37, 38]. This survey was designed to provide a high-quality public database for scientific research on this population. The survey used a multi-stage sampling method with strict randomization procedures to ensure sample representativeness. The county and rural administrative units were sampled for this study by using a probability proportional to size (PPS) sampling method [39].

### Statistical analysis

Cross-sectional study design is applied. Descriptive statistics were applied to analyze the features of the samples. We used univariate general linear regression models to compare the SRH score means across groups with different individual and provincial variables, and multilevel models were used with a mixed model procedure to explain the hierarchical structure of the data, with individuals (Level 1) nested within provinces (Level 2). We fitted the 2-level linear regression models with random intercepts and fixed slopes to test the correlation between HCBSs utilization and SRH among Chronically ill older adults. Additionally, we explored the association between the use of the three types of HCBSs (health care, social support, and daily care services) and SRH. We conducted SPSS version 26.0 for data analysis and set the significance level at *p*-value < 0.05.

## Results

Basic characteristics of 8,623 respondents are presented in Table 1. The mean score of SRH of the respondents was 3.19, of which 20.55% (n = 1,772) utilized HCBSs, 19.47% utilized health care services, 2.44% utilized social support services, only 0.55% utilized daily care services. The largest demographics were female (n = 4,492, 52.09%), aged 60–69 (n = 5,187, 60.15%), partnered (n = 6,842, 79.35%), with education of elementary school (n = 3,910, 45.34%), living in rural areas (n = 6,345, 73.58%), without income (n = 7,594, 88.07%), and without health insurance (n = 8,361, 96.96%). Furthermore, there were significant numbers for respondents who did not drink (n = 6,014, 69.74%) or smoke (n = 6,429, 74.56%), who exercised (n = 7,604, 88.18%), and who possessed three or more chronic diseases (n = 4,343, 50.37%). Most respondents lived in the provinces, within the third quartile of GDP per capita (n = 2,675, 31.02%) and in provinces with the second quartile of hospital beds per 10,000 persons (n = 2,310, 26.79%).

Better SRH was more prevalent among those who used HCBSs, were male, were aged 60–70, had a partner, had high educational levels, lived in an urban area, had an income, had one chronic disease, and who smoked, drank, or exercised. For the province-level characteristics, older adults with chronic diseases living in provinces with a GDP per capita in the first quartile and those with hospital beds per 10,000 persons in the second quartile had better SRH (Table 1).

**Table 1:** The SRH by individual- and province-level variables among Chinese older adults with chronic diseases in 2018

Variable	N (%)	Mean (SE)	95%CI for mean	Univariate Regression β	*p-*value
**Total**	8,623 (100)	3.19 (0.0105)	3.17, 3.21		
**Utilization of HCBSs**					
Yes	1,772 (20.55)	3.12 (0.0238)	3.07, 3.17	ref	
No	6,851 (79.45)	3.20 (0.0117)	3.18, 3.23	0.034	**0.001** ^**^
**Daily Care**					
Yes	47 (0.55)	3.00 (0.1427)	2.71, 3.29	ref	
No	8,576 (99.45)	3.19 (0.0105)	3.17, 3.21	0.014	0.189
**Health Care**					
Yes	1,679 (19.47)	3.14 (0.0243)	3.09, 3.19	ref	
No	6,944 (80.53)	3.20 (0.0116)	3.18, 3.22	0.024	**0.024** ^*^
**Social Support**					
Yes	210 (2.44)	2.85 (0.0676)	2.72, 2.99	ref	
No	8,413 (97.56)	3.19 (0.0106)	3.17, 3.22	0.054	**< 0.001** ^***^
**Gender**					
Male	4,131 (47.91)	3.13 (0.0152)	3.10, 3.16	ref	
Female	4,492 (52.09)	3.24 (0.0144)	3.21, 3.27	0.056	**< 0.001** ^***^
**Age**					
60-	5,187 (60.15)	3.15 (0.0136)	3.12, 3.18	ref	
70-	2,711 (31.44)	3.25 (0.0183)	3.22, 3.29	0.049	**< 0.001** ^***^
80-	725 (8.41)	3.21 (0.0362)	3.14, 3.28	0.017	0.126
**Marital status**					
Partnered	6,842 (79.35)	3.16 (0.0117)	3.14, 3.19	ref	
Single	1,781 (20.65)	3.27 (0.0233)	3.22, 3.31	0.043	**< 0.001** ^***^
**Education**					
Illiterate	2,558 (29.66)	3.29 (0.0200)	3.25, 3.33	ref	
Elementary school	3,910 (45.34)	3.21 (0.0153)	3.18, 3.24	0.038	**0.001** ^***^
Middle school	1,332 (15.45)	3.03 (0.0260)	2.98, 3.08	-0.065	**< 0.001** ^***^
High school or higher	823 (9.54)	3.01 (0.0319)	2.95, 3.07	-0.060	**< 0.001** ^***^
**Residence**					
Urban	1,691 (19.61)	3.02 (0.0228)	2.98, 3.07	ref	
Urban-rural continuum	587 (6.81)	3.12 (0.0402)	3.04, 3.20	0.026	**0.029** ^*^
Rural	6,345 (73.58)	3.24 (0.0123)	3.21, 3.26	0.097	**< 0.001** ^***^
**Income**					
Yes	1,029 (11.93)	2.88 (0.0293)	2.82, 2.94	ref	
No	7,594 (88.07)	3.23 (0.0111)	3.21, 3.25	0.115	**< 0.001** ^***^
**Health insurance**					
Yes	262 (3.04)	3.17 (0.0605)	3.05, 3.29	ref	
No	8,361 (96.96)	3.19 (0.0107)	3.17, 3.21	0.003	0.757
**Number of chronic diseases**					
1	2,163 (25.08)	2.78 (0.0205)	2.74, 2.82	ref	
2	2,117 (24.55)	3.00 (0.0204)	2.96, 3.04	0.101	**< 0.001** ^***^
≥ 3	4,343 (50.37)	3.48 (0.0137)	3.45, 3.51	0.361	**< 0.001** ^***^
**Smoking**					
Yes	2,194 (25.44)	3.12 (0.0204)	3.08, 3.16	ref	
No	6,429 (74.56)	3.21 (0.0122)	3.18, 3.23	0.037	**0.001** ^***^
**Drinking**					
Yes	2,609 (30.26)	3.02 (0.0187)	2.98, 3.05	ref	
No	6,014 (69.74)	3.26 (0.0125)	3.23, 3.28	0.114	**< 0.001** ^***^
**Exercising**					
Yes	7,604 (88.18)	3.15 (0.0110)	3.13, 3.17	ref	
No	1,019 (11.82)	3.46 (0.0321)	3.40, 3.53	0.104	**< 0.001** ^***^
**GDP per capita**					
Q1	1,878 (21.78)	3.28 (0.0230)	3.23, 3.32	ref	
Q2	2,590 (30.04)	3.26 (0.0178)	3.22, 3.29	0.058	**< 0.001** ^***^
Q3	2,675 (31.02)	3.14 (0.0195)	3.10, 3.18	0.054	**< 0.001** ^***^
Q4	1,480 (17.16)	3.02 (0.0252)	2.97, 3.07	-0.046	**< 0.001** ^***^
**Hospital beds per 10,000 persons**					
Q1	2,310 (26.79)	3.21 (0.0201)	3.17, 3.25	ref	
Q2	2,316 (26.86)	3.09 (0.0215)	3.05, 3.13	0.054	**< 0.001** ^***^
Q3	1,920 (22.27)	3.19 (0.0227)	3.15, 3.24	0.045	**< 0.001** ^***^
Q4	2,077 (24.07)	3.26 (0.0193)	3.22, 3.30	0.076	**< 0.001** ^***^

*Note*: HCBSs, home and community-based services; SE, standard error; CI confidence interval

*p < 0.05, **p < 0.01, ***p < 0.001, bold emphasis indicates statistical significance

Table 2 summarizes the association between HCBSs utilization and SRH in older adults with chronic diseases. It turned out that SRH among older adults with chronic diseases was greatly associated with HCBSs utilization (β = 0.085, SE = 0.025, *p* < 0.001, 95% CI = 0.037–0.133) after adjusting for individual-level and province-level variables. The indexes of -2 log likelihood, AIC and BIC are used to represent the model fit, being 22757.454, 22761.454 and 22775.572, respectively.

Supplementary Tables 1–3 show the relationship between the three categories of HCBSs use and SRH among Chinese older adults with chronic diseases separately. SRH among older adults with chronic diseases was strongly linked to the use of health care and social support services (β = 0.068, SE = 0.025, p < 0.001; β = 0.239, SE = 0.063, *p* < 0.001, respectively) after adjusting for individual-level and province-level variables. However, there was no significant association between the use of daily care services and SRH among older adults with chronic diseases.

**Table 2:** The use of HCBSs associated with SRH among Chinese old adults with chronic diseases in 2018

Variables	β (SE)	95% CI	
**Fixed effects**			
**Individual level**			
Intercept	2.333 (0.102)^***^	2.130	2.535
HCBSs (ref: yes)	0.085 (0.025)^**^	0.037	0.133
Gender (ref: male)	-0.021 (0.025)	-0.070	0.028
Age (ref: 60-)			
70-	0.013 (0.022)	-0.030	0.057
80-	-0.055 (0.038)	-0.130	0.019
Marital (ref: partnered)	0.036 (0.026)	-0.014	0.086
Education (ref: illiterate)			
Elementary school	-0.039 (0.025)	-0.088	0.010
Middle school	-0.136 (0.034)^***^	-0.203	-0.069
High school or higher	-0.118 (0.041)^**^	-0.198	-0.038
Residence (ref: urban)			
Urban-Rural continuum	0.086 (0.044)^*^	0.001	0.171
Rural	0.197 (0.027)^***^	0.143	0.250
Income (ref: yes)	0.196 (0.031)^***^	0.135	0.257
Health insurance (ref: yes)	0.104 (0.057)	-0.009	0.216
Number of chronic diseases (ref: 1)			
2	0.216 (0.028)^***^	0.0162	0.270
≥ 3	0.689 (0.024)^***^	0.641	0.736
Smoking (ref: yes)	-0.033 (0.026)	-0.083	0.018
Drinking (ref: yes)	0.145 (0.023)^***^	0.099	0.191
Exercising (ref: yes)	0.240 (0.031)^***^	0.179	0.300
**Province level**			
GDP per capita (ref: Q1)			
Q2	0.013 (0.074)	-0.141	0.167
Q3	-0.106 (0.072)	-0.257	0.045
Q4	-0.171 (0.082)^*^	-0.343	-0.001
Number of beds in medical institutions per 10,000 persons (ref: Q1)			
Q2	-0.042 (0.072)	-0.195	0.110
Q3	-0.070 (0.076)	-0.230	0.090
Q4	0.030 (0.079)	-0.137	0.196
**Random effect**			
Repeated	0.807 (0.012)^***^		
Intercept (province)	0.014 (0.006)^*^		
**Model fit**			
-2 log likelihood	22757.454		
AIC	22761.454		
BIC	22775.572		

*Note*: HCBSs, home and community-based services; AIC: Akaike’s Information Criterion; BIC: Schwarz’s Bayesian Criterion; β, regression coefficient; SE, standard error; *p < 0.05, **p < 0.01, ***p < 0.001

## Discussion

To the best of our knowledge, this is the first study to examine the relationship between the use of HCBSs and SRH in chronically ill older adults in China. Further, this study evaluated the roles of individual- and provincial-level covariates in SRH among chronically ill older adults. The results showed that chronically ill older adults who used HCBSs have an increased likelihood of having better SRH, even after adjusting for individual- and province-level covariates.

Previous studies of SRH among older adults have usually focused only on individual factors or provincial factors, leading to scientific bias in the research. In this study, the hierarchical linear regression model was used to comprehensively consider the implication of individual and provincial factors, which made results of the study more reliable. In addition, the CHALRS database applied multi-stage sampling approach with rigorous randomization procedures. All the respondents were randomly assigned, and their exclusion could not influence the robustness of the results [40, 41]. The results of this study are in line with the previous studies, finding that individual factors including education [42], residence [43], income [44], number of chronic diseases [45], drinking [46], and exercising [46], were probably linked to SRH among Chinese older adults with chronic diseases.

This study concludes that HCBSs utilization may have a facilitative effect on the SRH among chronically ill older adults. They tend to have poorer health status, worse mobility, and greater social isolation than the general elderly population [47]. Depending on the features of chronically ill older adults, HCBSs have a targeted role to play. **Firstly**, HCBSs offer proximity and save time and money, which are partially significant in promoting physical health in chronically ill older adults, and better meet the demand for health care services, thus promoting their SRH [48, 49]. **Secondly**, HCBSs contribute to improving the mental health of chronically ill older adults by encouraging them to participate in social activities as well as meeting their interpersonal communication needs, which can make them feel protected and respected, promoting their SRH [50, 51]. **Thirdly**, HCBSs may keep chronically ill older adults out of hospital and institutions and provide adequate assistance in primary activities (bathing, dressing, eating, toilet visit, getting in and out of bed, and moving around the room) [52]. Consequently, they will feel they are cared for, giving rise to better SRH. Overall, HCBSs utilization was positively related to SRH among chronically ill older adults.

To further analyze the relationship between HBCSs utilization and SRH in older adults with chronic diseases, this study explored the relationship with SRH with greater precision by dividing HCBSs into health care, daily care, and social support services. The results indicated that the use of health care and social support services was closely linked to SRH while that of daily care services has no link with SRH. Theoretically, all three types of services can promote SRH in older adults with chronic diseases [53–55]. Although the utilization rate of health care services is relatively reasonable at 19.47%, reflecting the finding of this study [56]. However, the results of the relationship between daily care and social support services and SRH are biased by the fact that the utilization rates (0.55% and 2.44% respectively) are extremely low that the finding must be treated with care. Additionally, studies have shown that the demand rates for social support services and daily care services in HCBSs were over 60% in 2018 in China, whereas the utilization rate was extremely low [57]. This study supposed that the low utilization of social support and daily care services might be due to a mismatch between supply and demand as a result of the lack of response to demand for various services among chronically ill older adults [58].

Although chronically ill older adults are a key focus of China’s elderly policy and a crucial target of HCBSs [15], the actual implementation of the policy has revealed a considerably low utilization of daily care and social support services, probably due to a lack of supply of services, poor quality of services and insufficient promotion of services. **First of all**, one study has shown that the supply rates of health care, social support and daily care services in HCBSs in China were 53.8%, 40.3% and 15.7% respectively in 2018 [46], indicating insufficient supply for older adults with chronic diseases to take advantage of the services. **In addition**, older adults with chronic diseases may have higher requirements for long-term ongoing care and spiritual comfort than the general elderly population, thus higher service quality is required. Nevertheless social support and daily care services provided by the community cannot meet the needs of chronically ill older adults on account of the unprofessional nature of the service staff and the unregulated relevant service process, leading to many services facilities being idle and experiencing low utilization rates [55]. **Eventually**, older adults with chronic diseases (e.g. hypertension, diabetes and coronary heart disease) require regular medical check-ups and in-home medical visits, and are therefore likely to be more aware of community-based health care services than daily care and social support services. This, coupled with a lack of necessary publicity for these daily care and social support services in the community, may result in low utilization of the services [12, 55].

In terms of the supply of daily care and social support services, the government should start from the demand side by conducting research to understand the needs of older adults with chronic diseases for the corresponding services, and providing targeted services and improving the supply of services; secondly, the government should improve the quality of services by encouraging and supporting diversified entities to ensure the long-term continuity of care for older adults with chronic diseases, reducing the number of visits to the doctor; Ultimately, the government should increase health talks on community-based chronic disease care, and the safe use of exercise facilities to raise awareness of daily care and social support services among chronically ill older adults.

## Conclusions

This study showed that HCBSs utilization was linked to SRH in older adults with chronic diseases in China. Of the HCBSs, the utilization rate of health care services was the highest. The government should provide targeted health care services to chronically ill older adults and promote their SRH. Regarding daily care and social support, the government should focus on chronically ill older adults, increase the supply of services, improve the quality of services, and enhance publicity about the services so that chronically ill older adults can age and live well in the community.

### Limitation

There are several limitations to this study. Above all, the causal relationship between HCBSs utilization and SRH in chronically ill older adults was not examined. Prospective studies should examine the relationship. Second, This study only considered the health dimension of SRH and did not separately examine the relationship between HCBSs utilization and physical health and mental health. Third, the CHARLS database only includes the main services currently provided by HCBSs in China. In recent years, China’s HCBSs have developed rapidly, and, in some areas, services such as legal advocacy, psychological counseling, and rehabilitation care have been added. Subsequent surveys should add relevant categories.

### Electronic supplementary material

Below is the link to the electronic supplementary material.

Supplementary Material 1

Supplementary Material 2

## Data Availability

The data are available at http://charls.pku.edu.cn.

## Abbreviations

HCBSs: home and community-based services
SRH: self-rated health
CHARLS: China Health and Retirement Longitudinal Study
