# Supplementary material for: Association between home and community-based services utilization and self-rated health among Chinese older adults with chronic diseases: evidence from the 2018 China Health and Retirement Longitudinal Study
Source: BMC Public Health. 2024 Jan 8;24:117. doi: 10.1186/s12889-023-17535-1 (PMC10775520; doi:10.1186/s12889-023-17535-1)
Supplement: Supplementary file 2 — Supplementary Material 2 [file 12889_2023_17535_MOESM2_ESM.docx]

Supplementary Table 1. The use of health care services associated with SRH among Chinese old adults with chronic diseases in 2018.

| **Variables** | ***β*(*SE*)** | **95% C.I** | |
| --- | --- | --- | --- |
| **Fixed effects** |  |  |  |
| **Individual level** |  |  |  |
| Intercept | 2.349 (0.102)^***^ | 2.146 | 2.552 |
| HCBSs in health care (ref: yes) | 0.068 (0.025)^**^ | 0.018 | 0.117 |
| Gender (ref: male) | -0.021 (0.025) | -0.071 | 0.028 |
| Age (ref: 60-) |  |  |  |
| 70- | 0.012 (0.022) | -0.032 | 0.056 |
| 80- | -0.057 (0.038) | -0.131 | 0.018 |
| Marital (ref: partnered) | 0.035 (0.026) | -0.015 | 0.085 |
| Education (ref: illiterate) |  |  |  |
| Elementary school | -0.040 (0.025) | -0.089 | 0.009 |
| Middle school | -0.137 (0.034)^***^ | -0.204 | -0.070 |
| High school or higher | -0.120 (0.119)^**^ | -0.199 | -0.039 |
| Residence (ref: urban) |  |  |  |
| Urban-Rural continuum | 0.087 (0.044)^*^ | 0.002 | 0.172 |
| Rural | 0.197 (0.027)^***^ | 0.144 | 0.251 |
| Income (ref: yes) | 0.196 (0.031)^***^ | 0.134 | 0.257 |
| Health insurance (ref: yes) | 0.102 (0.057) | -0.010 | 0.214 |
| Number of chronic diseases (ref: 1) |  |  |  |
| 2 | 0.216 (0.028)^***^ | 0.162 | 0.270 |
| ≥3 | 0.689 (0.024)^***^ | 0.641 | 0.736 |
| Smoking (ref: yes) | -0.033 (0.026) | -0.084 | 0.018 |
| Drinking (ref: yes) | 0.146 (0.023)^***^ | 0.100 | 0.192 |
| Exercising (ref: yes) | 0.241 (0.031)^***^ | 0.180 | 0.301 |
| **Province level** |  |  |  |
| GDP per capita (ref: Q1) |  |  |  |
| Q2 | 0.013 (0.073) | -0.141 | 0.168 |
| Q3 | -0.105 (0.072) | -0.256 | 0.046 |
| Q4 | -0.171 (0.082) | -0.343 | 0.000 |
| Number of beds in medical institutions per 10,000 persons (ref: Q1) |  |  |  |
| Q2 | -0.044 (0.072) | -0.196 | 0.109 |
| Q3 | -0.070 (0.076) | -0.230 | 0.090 |
| Q4 | 0.029 (0.079) | -0.138 | 0.195 |
| **Random effect** |  |  |  |
| Repeated | 0.807 (0.012)^***^ |  |  |
| Intercept (province) | 0.014 (0.006)^*^ |  |  |
| **Model fit** |  |  |  |
| -2 log likelihood | 22762.209 |  |  |
| AIC | 22766.209 |  |  |
| BIC | 22780.328 |  |  |

Note. AIC: Akaike’ s Information Criterion; BIC: Schwarz’ s Bayesian Criterion; β, regression coefficient; SE, standard error; *p<0.05, **p<0.01, ***p<0.001.

Supplementary Table 2. The use of daily care services associated with SRH among Chinese old adults with chronic diseases in 2018.

| **Variables** | ***β*(*SE*)** | **95% C.I** | |
| --- | --- | --- | --- |
| **Fixed effects** |  |  |  |
| **Individual level** |  |  |  |
| Intercept | 2.177 (0.166)^***^ | 1.851 | 2.503 |
| HCBSs in daily care (ref: yes) | 0.236 (0.132) | -0.023 | 0.495 |
| Gender (ref: male) | -0.022 (0.025) | -0.071 | 0.027 |
| Age (ref: 60-) |  |  |  |
| 70- | 0.008 (0.022) | -0.035 | 0.052 |
| 80- | -0.060 (0.038) | -0.135 | 0.015 |
| Marital (ref: partnered) | 0.036 (0.026) | -0.015 | 0.086 |
| Education (ref: illiterate) |  |  |  |
| Elementary school | -0.043 (0.025) | -0.092 | 0.006 |
| Middle school | -0.138 (0.034)^***^ | -0.205 | -0.071 |
| High school or higher | -0.121 (0.041)^**^ | -0.201 | -0.040 |
| Residence (ref: urban) |  |  |  |
| Urban-Rural continuum | 0.089 (0.044)^*^ | 0.003 | 0.174 |
| Rural | 0.198 (0.027)^***^ | 0.144 | 0.251 |
| Income (ref: yes) | 0.195 (0.031)^***^ | 0.134 | 0.257 |
| Health insurance (ref: yes) | 0.099 (0.057) | -0.013 | 0.211 |
| Number of chronic diseases (ref: 1) |  |  |  |
| 2 | 0.214 (0.028)^***^ | 0.160 | 0.268 |
| ≥3 | 0.686 (0.024)^***^ | 0.638 | 0.733 |
| Smoking (ref: yes) | -0.035 (0.026) | -0.085 | 0.016 |
| Drinking (ref: yes) | 0.146 (0.023)^***^ | 0.100 | 0.192 |
| Exercising (ref: yes) | 0.246 (0.031)^***^ | 0.185 | 0.306 |
| **Province level** |  |  |  |
| GDP per capita (ref: Q1) |  |  |  |
| Q2 | 0.016 (0.074) | -0.139 | 0.171 |
| Q3 | -0.104 (0.072) | -0.257 | 0.048 |
| Q4 | -0.171 (0.082) | -0.344 | 0.001 |
| Number of beds in medical institutions per 10,000 persons (ref: Q1) |  |  |  |
| Q2 | -0.049 (0.073) | -0.202 | 0.105 |
| Q3 | -0.067 (0.077) | -0.228 | 0.095 |
| Q4 | 0.026 (0.080) | -0.141 | 0.194 |
| **Random effect** |  |  |  |
| Repeated | 0.807 (0.012)^***^ |  |  |
| Intercept (province) | 0.014 (0.006)^*^ |  |  |
| **Model fit** |  |  |  |
| -2 log likelihood | 22762.959 |  |  |
| AIC | 22766.959 |  |  |
| BIC | 22781.077 |  |  |

Note. AIC: Akaike’ s Information Criterion; BIC: Schwarz’ s Bayesian Criterion; β, regression coefficient; SE, standard error; *p<0.05, **p<0.01, ***p<0.001.

Supplementary Table 3. The use of social support services associated with SRH among Chinese old adults with chronic diseases in 2018.

| **Variables** | ***β*(*SE*)** | **95% C.I** | |
| --- | --- | --- | --- |
| **Fixed effects** |  |  |  |
| **Individual level** |  |  |  |
| Intercept | 2.178 (0.117)^***^ | 1.946 | 2.409 |
| HCBSs in social support (ref: yes) | 0.239 (0.063)^***^ | 0.115 | 0.363 |
| Gender (ref: male) | -0.021 (0.025) | -0.071 | 0.028 |
| Age (ref: 60-) |  |  |  |
| 70- | 0.008 (0.022) | -0.035 | 0.052 |
| 80- | -0.063 (0.038) | -0.138 | 0.012 |
| Marital (ref: partnered) | 0.036 (0.026) | -0.014 | 0.086 |
| Education (ref: illiterate) |  |  |  |
| Elementary school | -0.041 (0.025) | -0.090 | 0.008 |
| Middle school | -0.134 (0.034)^***^ | -0.201 | -0.067 |
| High school or higher | -0.116 (0.041)^**^ | -0.197 | -0.036 |
| Residence (ref: urban) |  |  |  |
| Urban-Rural continuum | 0.090 (0.044)^*^ | 0.005 | 0.175 |
| Rural | 0.197 (0.027)^***^ | 0.143 | 0.250 |
| Income (ref: yes) | 0.196 (0.031)^***^ | 0.135 | 0.257 |
| Health insurance (ref: yes) | 0.100 (0.057) | -0.012 | 0.212 |
| Number of chronic diseases (ref: 1) |  |  |  |
| 2 | 0.212 (0.028)^***^ | 0.158 | 0.266 |
| ≥3 | 0.685 (0.024)^***^ | 0.637 | 0.732 |
| Smoking (ref: yes) | -0.034 (0.026)^***^ | -0.084 | 0.017 |
| Drinking (ref: yes) | 0.144 (0.023)^***^ | 0.098 | 0.190 |
| Exercising (ref: yes) | 0.244 (0.031)^***^ | 0.183 | 0.304 |
| **Province level** |  |  |  |
| GDP per capita (ref: Q1) |  |  |  |
| Q2 | 0.014 (0.074) | -0.141 | 0.168 |
| Q3 | -0.104 (0.072) | -0.255 | 0.048 |
| Q4 | -0.170 (0.082) | -0.342 | 0.002 |
| Number of beds in medical institutions per 10,000 persons (ref: Q1) |  |  |  |
| Q2 | -0.047 (0.073) | -0.200 | 0.105 |
| Q3 | -0.065 (0.076) | -0.226 | 0.095 |
| Q4 | 0.027 (0.079) | -0.139 | 0.194 |
| **Random effect** |  |  |  |
| Repeated | 0.806 (0.012)^***^ |  |  |
| Intercept (province) | 0.014 (0.006)^*^ |  |  |
| **Model fit** |  |  |  |
| -2 log likelihood | 22753.348 |  |  |
| AIC | 22757.348 |  |  |
| BIC | 22771.466 |  |  |

Note. AIC: Akaike’ s Information Criterion; BIC: Schwarz’ s Bayesian Criterion; β, regression coefficient; SE, standard error; *p<0.05, **p<0.01, ***p<0.001.

**Supplementary Table 4.** STROBE Statement of this study.

|  | Item No | Recommendation |
| --- | --- | --- |
| **Title and abstract** | 1 | (*a*) Association between home and community-based services utilization and self-rated health among Chinese older adults with chronic diseases: Evidence from the 2018 China Health and Retirement Longitudinal Study |
|  |  | (*b*) line 4-31, page 3-4 |
| Introduction | | |
| Background/rationale | 2 | Line 34-86, page 4-6 |
| Objectives | 3 | Line 87-92, page 6-7 |
| Methods | | |
| Study design | 4 | Line 96-100, page 7 |
| Setting | 5 | Line 96, page 7 |
| Participants | 6 | Line 102-105, page 7 |
| Variables | 7 | Line 107-129, page 7-8 |
| Data sources/ measurement | 8* | Line 132-138, page 8-9 |
| Bias | 9 | The missing variables were missing at complete random, their exclusion therefore did not affect the robustness of the results. |
| Study size | 10 | Line 102-105, page 7 |
| Quantitative variables | 11 | Line 102-105, page 7 |
| Statistical methods | 12 | Line 139-148, page 9 |
| **Results** | | |
| Participants | 13* | Line 150-151, page 9 |
| Descriptive data | 14* | Line 150-163, page 9-10 |
| Outcome data | 15* | Line 164-169, page 10 |
| Main results | 16 | Line 172-184, page 15 |
| Other analyses | 17 | Not applicable |
| **Discussion** | | |
| Key results | 18 | Line 192-197, page 17 |
| Limitations | 19 | Line 275-283, page 20-21 |
| Interpretation | 20 | Line 207-263, page 17-20 |
| Generalisability | 21 | Line 198-206, page 17 |
| **Other information** | | |
| Funding | 22 | Line 302-307, page 22 |

*Give information separately for cases and controls in case-control studies and, if applicable, for exposed and unexposed groups in cohort and cross-sectional studies.

**Note:** An Explanation and Elaboration article discusses each checklist item and gives methodological background and published examples of transparent reporting. The STROBE checklist is best used in conjunction with this article (freely available on the Web sites of PLoS Medicine at http://www.plosmedicine.org/, Annals of Internal Medicine at http://www.annals.org/, and Epidemiology at http://www.epidem.com/). Information on the STROBE Initiative is available at www.strobe-statement.org.
